# Bitter Melon Extract Yields Multiple Effects on Intestinal Epithelial Cells and Likely Contributes to Anti-diabetic Functions

**DOI:** 10.7150/ijms.55866

**Published:** 2021-02-24

**Authors:** Chi-I Chang, Shi-Yie Cheng, Annisa Oktafianti Nurlatifah, Wei-Wen Sung, Jing-Hong Tu, Lin-Lee Lee, Hsueh-Ling Cheng

**Affiliations:** 1 Department of Biological Science and Technology, National Pingtung University of Science and Technology, Pingtung 91201, Taiwan.; 2 Department of Life Sciences, National University of Kaohsiung, Kaohsiung 811, Taiwan.; 3 Department of Agroindustrial Biotechnology, Brawijaya University, Jalan, Veteran Malang 65145, Indonesia.; 4 Department of English, National Kaohsiung Normal University, Kaohsiung 80201, Taiwan.

**Keywords:** AMP-activated protein kinase, bitter-taste receptor, glucagon-like peptide 1, diabetes, intestine, *Momordica charantia.*

## Abstract

The intestines have been recognized as important tissues for metabolic regulation, including glycemic control, but their vital role in promoting the anti-diabetic effects of bitter melon, the fruit of *Momordica charantia* L, has seldom been characterized, nor acknowledged. Evidence suggests that bitter melon constituents can have substantial interactions with the intestinal epithelial cells before circulating to other tissues. We therefore characterized the effects of bitter melon extract (BME) on intestinal epithelial cells. BME was found to contain substantial amounts of carbohydrates, proteins, and triterpenoids. TNF-α induced insulin resistance in an enterocyte cell line of IEC-18 cells, and BME promoted glucose utilization of the insulin-resistant cells. Further analysis suggested that the increased glucose consumption was a result of the combined effects of insulin sensitizing and insulin substitution functions of BME. The functions of insulin substitution were likely generated due to the activation of AMP-activated protein kinase. Meanwhile, BME acted as a glucagon-like peptide 1 (GLP-1) secretagogue on enteroendocrine cells, which may be mediated by the activation of bitter-taste receptors. Therefore, BME possesses insulin sensitizing, insulin substitution, and GLP-1 secretagogue functions upon intestinal cells. These effects of BME on intestinal cells likely play a significant part in the anti-diabetic action of bitter melon.

## Introduction

Diabetes, a chronic disorder characterized by hyperglycemia, is classified into several types, among which type 1 and type 2 diabetes are the most prevalent. Type 1 diabetes, caused by low or no insulin secretion from impaired pancreatic β cells, likely results from autoimmune problems. Type 2 diabetes is initiated by insulin resistance, suggesting insulin-sensitive tissues cannot respond to insulin stimulation properly, and usually resulting in progressive β-cell dysfunction and insufficient insulin secretion 1, 2.

The whole plant of *Momordica charantia* L. has been reported to have anti-diabetic functions 3-7, whereas bitter melon (also known as bitter gourds or balsam pears), i.e., the fruit of *M. charantia* L., are the most commonly consumed parts of the plant as food or herbal medicine. Therefore, we attempted to analyze the lesser-known effects of bitter melon on the intestines regarding glycemic control, in view of the role of the intestines in metabolic regulation including the glycemia, but noting the scarcity of reference to their role in the anti-diabetic effects of bitter melon.

The mechanisms underlying the hypoglycemic effect of bitter melon have been widely studied and explored in insulin-sensitive tissues, but mostly in the liver, the skeletal muscles and the adipose tissues 4, 5, 8, 9. However, a previous study discovered that triterpenoids purified from bitter melon were transported across an intestinal epithelial cell monolayer *in vitro*, and small amounts of these triterpenoids stayed inside the epithelial cells 10. Thus, bitter melon' natural compounds theoretically can have substantial interactions with the intestinal epithelial cells prior to working on other tissues. Moreover, the concentrations of bitter melon compounds encountered by the intestinal epithelial cells *in situ* during nutrient absorption from the lumen are likely to be higher than those encountered by other organs through circulation. However, other than the inhibition of intestinal α-amylase and α-glucosidase, and the suppression of intestinal glucose absorption from the digestive lumen 11, 12, the effects of bitter melon components on intestinal cells were rarely characterized.

Intestinal cells are also sensitive to insulin and can develop insulin resistance in obese or inflammatory conditions 13-16. Additionally, the intestines play important roles in metabolism regulation, including glycemic control, since they secrete several hormones to regulate metabolism while being stimulated by food components or metabolic changes 17. Meanwhile, the intestines occupy a substantial mass of the human body and carry out intensive physiological activities and as such are important vectors in blood glucose utilization, contributing to the reduction of glycemia 13, 18. Consequently, the responses of intestinal epithelial cells to bitter melon constituents should be carefully re-examined.

The epithelial layer of the intestines is composed of several cell types, including columnar enterocytes in the majority, and enteroendocrine cells that secrete several hormones upon stimulation. The enterocytes were reported to be insulin sensitive 13-15, 19, whereas the enteroendocrine cells were demonstrated to contain bitter-taste receptors (TAS2Rs), a group of G protein-coupled receptors classified as taste receptor 2 family 20. The activation of TAS2Rs by bitter substances provokes secretion of several hormones from enteroendocrine cells, including glucagon-like peptide 1 (GLP-1) 21. GLP-1, a notable target for developing anti-diabetic medicines, regulates the glycemic level through several mechanisms, such as the control of insulin secretion 21-23. The activation of TAS2Rs on enteroendocrine cells triggers the dissociation of β and γ subunits from the α subunit of the coupled trimeric G proteins. The Gβγ complex activates phospholipase C β_2_ (PLCβ_2_), which catalyzes the production of inositol-1,4,5-triphosphate (IP3) and diacylglycerol from phosphatidylinositol-4,5-diphosphate in the cell membrane. IP3 activates IP3 receptors in the membrane of the endoplasmic reticulum, resulting in the release of Ca^2+^ from the endoplasmic reticulum, and thus an increase in cytosolic [Ca^2+^]. Subsequently, through a not completely clarified mechanism, the increased cytosolic [Ca^2+^] leads to the secretion of GLP-1 from the enteroendocrine cells 24-26.

The constituents of bitter melon have been demonstrated to exert effects on insulin-sensitive cells 4, 8, and bitterness is a characteristic taste of this vegetable. Thus, we analyzed the responses of enterocytes and enteroendocrine cells to bitter melon extract (BME) in order to evaluate the effects of bitter melon on the intestines.

## Materials and methods

### Reagents

Antibodies for total or phosphorylated Akt (Ser 473) and total or phosphorylated AMPK α subunit (Thr 172) were purchased from Cell Signaling Technology (Beverley, MA, USA); horseradish peroxidase-conjugated secondary antibodies were from Santa Cruz Biotechnologies (Santa Cruz, CA, USA). Fetal bovine serum (FBS) was acquired from Invitrogen (Carlsbad, CA, USA). Protease inhibitor cocktail and phosphatase inhibitor cocktail were from Calbiochem (Merck Millipore, Darmstadt, Germany). Cell Culture Lysis Reagent was from Promega (Madison, WI, USA). Folin-Ciocalteau reagent was procured from Fluka Biochemica (Buchs, Switzerland), whereas methanol and ethanol were purchased from Merck (Darmstadt, Germany). Cell culture media, bovine insulin solution, dimethylsulfoxide (DMSO), mouse TNF-α, rosiglitazone, denatonium benzoate, Fluo-4 AM, Compound C, gallein, U73122, and other chemicals were ordered from Sigma Chemical Company (St. Louis, MO, USA).

### Preparation and chemical composition analysis of bitter melon extract

Bitter melon (the fruit of *M. charantia* L.) were collected from a local farm in Pingtung County, Taiwan, in July 2018. A voucher specimen (MC) has been identified by Prof. Sheng-Zehn Yang, Curator of Herbarium, National Pingtung University of Science and Technology and deposited in the Department of Life Science, National University of Kaohsiung. The sample was oven-dried (53^o^ C) for one week. The dried specimen (1.0 kg, dry weight) was chopped into small pieces and extracted three times with ethanol (3 × 1.5 L, 2 days each) in a percolator at room temperature. After filtration and solvent evaporation, the combined ethanol extracts were concentrated in vacuo (under 35^o^ C) to afford a resulting light yellow gum (35.0 g), i.e., BME. Part of BME was dissolved in DMSO, and subjected to protein concentration assay by using Bradford reagent (Bio-Rad, Hercules, CA, USA), as well as to total carbohydrate assay by using the phenol-sulfuric acid method 27.

The total phenolic content of BME was determined using the Folin-Ciocalteu assay with some modifications 28. Briefly, 25 μL of BME solutions (50-1000 μg/mL in methanol) were mixed with 100 μL of 20% Na_2_CO_3_. After 2 min, 50 μL of 20% Folin-Ciocalteau reagent was subsequently added to the mixture and then incubated for 30 min at room temperature. The absorbance of the reaction mixtures was measured at 750 nm using a microplate spectrophotometer (Multiskan GO, Thermo Fischer Scientific, Vantaa, Finland). Gallic acid (2-200 μg/mL in methanol) was used as a standard to plot the calibration curve. The total phenolic contents of BME was expressed as mg gallic acid equivalents (GAE) per gram dry sample (mg GAE/g dry sample).

Total flavonoid content of BME was determined following the method described by Barreira et al. with some modifications 29. Briefly, 25 μL aliquots of BME solutions (125-1000 μg/mL in methanol) were mixed with 152.5μL of deionized water followed by an addition of 7.5 μL of 5% NaNO_2_. The mixture was allowed to stand at room temperature for 6 min and then 15 μL of a 10% AlCl_3_·6H2O solution was added into the mixture. After a further 5 min incubation at room temperature, 50 μL of 1M NaOH was added to the mixture. The mixture was then incubated for 15 min at room temperature. The absorbance at 500 nm was read using a microplate spectrophotometer. Quercetin (4-400 μg/mL in methanol) was used as a standard to draw the calibration curve. The total flavonoid content was expressed as mg quercetin equivalents (QE) per gram dry sample (mg QE/g dry sample).

The total triterpenoid content of BME was determined by the method of Fan and He 30 with a slight modification. Briefly, 100 μL of BME (10 mg/mL in methanol) was mixed with 150 μL of 5% (w/v) vanillin-glacial acetic acid solution and 500 μL perchloric acid solution. The mixture was incubated at 80°C for 30 min. Subsequently, the solution was cooled in an ice-water bath to the ambient temperature and diluted to 3 mL. Absorbance at 548 nm was measured using a microplate spectrophotometer and compared to standard ursolic acid calibration curve (25-500 μg/mL in methanol). Results were expressed as mg ursolic acid equivalents (UAE) per gram dry sample (mg UAE/g dry sample).

### Cell culture and cytotoxicity assays

NCI-H716 cells and IEC-18 cells were purchased from Bioresource Collection and Research Center (Hsinchu, Taiwan), and cultured at 37° C in a humidified incubator supplied with 5% CO_2_ using Dulbecco's modified Eagle's medium and RPMI 1640 medium, respectively, containing 10% FBS.

Cells were seeded in 96-well plates in a density of 1 x 10^4^ cells/well (IEC-18) or 1 x 10^6^ cells/well (NCI-H716), and treated in triplicate with the desired concentration of BME, or an equal volume of DMSO (solvent; as the control) in a serum-free medium for 6 h. Subsequently, a quick cell proliferation assay kit (BioVision, Milpitas, CA, USA) was added per the supplier's instructions, and the plates were incubated for 2 h at 37° C. Absorbance at 450 nm and 650 nm was measured afterwards. The relative survival rate versus the control was determined, and the mean ± standard error (SE) was calculated.

### Glucose uptake assays

Glucose uptake assays in normal IEC-18 cells were performed as previously described 19. For assays in insulin-resistant cells, IEC-18 cells were seeded equally in 96-well plates, cultured overnight, and incubated in a serum-free medium containing the desired concentration of TNF-α for 5 h to induce insulin resistance in the cells. The medium was removed, replaced with 50 μL fresh, serum-free medium containing 100 nM insulin, the desired concentration of BME, or an equal volume of the solvent (control), and incubated at 37° C for 5 h. Compound C was added 30 min earlier than the addition of BME when it was required. Each treatment was performed in triplicate. At 0 and 5 h after the treatment, 5 μL of the medium was withdrawn, mixed with 200 μL of a glucose assay kit (Glucose GOD FS, Diagnostic Systems, Holzheim, Germany), and reacted at 37° C for 10 min. Absorbance at 500 nm was detected in order to analyze the glucose concentration in the medium. The cell number in each well was subsequently analyzed by using the quick cell proliferation assay kit as described above. Glucose consumption by each well was determined based on the OD_500_ difference between 0 h and 5 h, and was normalized by cell number. Relative glucose uptake versus the control was then calculated.

### Western blot analysis

Cells were seeded in 35- or 60-mm plates, cultured until approximately 90% confluence, washed with phosphate-buffered saline (PBS; pH 7.4), and incubated in a serum-free medium containing 20 ng/mL of TNF-α for 5 h. After being washed twice with PBS, the cells were incubated in a serum-free medium containing 100 nM insulin, a desired concentration of BME, 50 μM rosiglitazone, or an equal volume of the solvent for 1 h. The cells were then washed twice with PBS, submerged in a lysis buffer (Cell Culture Lysis Reagent containing a protease inhibitor cocktail, and a phosphatase inhibitor cocktail), and scraped off the plate on ice. The resulting suspension was centrifuged at 12,000 × *g* for 10 min at 4°C. The supernatant was collected and analyzed for protein concentration using Bradford reagent. Equal amounts of proteins were sampled from different treatments, and subjected to electrophoresis and western blotting as described previously 31, 32. Immunoreactive bands were developed on the PVDF membrane using Supersignal West Femto Maximum Sensitivity Substrate (Thermo Scientific, Rockford, IL, USA) and detected using an imaging system (UVP BioSpectrum, UVP, LLC, Upland, CA, USA). The band intensities were determined using the supplied software.

### GLP-1 assays

NCI-H716 cells were seeded equally in 24-well plates precoated with Matrigel (Corning, Corning, NY, USA), cultured as described previously 19, and treated for 1 h with 200 μg/mL BME, 10 mM denatonium benzoate (dissolved in 4% DMSO), or the respective solvent in PBS containing 0.9 mM CaCl_2_. In cultures treated with an inhibitor, the inhibitor was added 30 min earlier than BME. GLP-1 concentration in the medium was then determined by using a GLP-1 ELISA kit (RayBiotech, Peachtree Corners, GA, USA).

### Calcium fluorescent imaging assay

NCI-H716 cells were seeded in clear-bottom 96-well black plates (Corning) precoated with Matrigel. Cells were then incubated with serum-free medium containing 4 μM Fluo-4 AM for 1 h. After being washed 3 times with PBS, the medium was replaced with Dulbecco's phosphate-buffered saline containing 2 mM calcium (DPBS). Cells were incubated in DPBS for 30 min at 37°C, washed 3 times with DPBS, and incubated in DPBS again. The plate was then placed under a fluorescent microscope (IX73, Olympus, Tokyo, Japan) equipped with an imaging system (DP80, Olympus) to start recording fluorescent imaging. BME, denatonium benzoate, or the solvent, was added to the saline, and the real-time intracellular fluorescence fluctuations were recorded by the imaging system and quantified with the supplied software (Dimension, Olympus). For experiments treated with an inhibitor, the inhibitor was added during the 30-min incubation with DPBS, and was present during fluorescence recording. The fold of fluorescence increment relative to the background was calculated as ΔF/F = (Fn - F_0_)/F_0_, in which F_0_ is the relative fluorescence unit obtained at time = 0 sec; Fn is the relative fluorescence unit recorded at time = n sec.

### Statistical analysis

Data were analyzed using one-way analysis of variance (ANOVA) followed by Scheffe's post hoc test. Significance was considered when *p* < 0.05 and F > 3.5546.

## Results

### Bitter melon extract promoting the glucose utilization of insulin-resistant enterocytes

The protein, carbohydrate, phenolic compound, flavonoid, and triterpenoid content of BME were analyzed. As a result, BME contains 5.46 ± 0.07 % (w/w) of total proteins and 17.20 ± 0.85% (w/w) of total carbohydrates. Total phenolic content, total flavonoids, and total triterpenoids in BME are 2.31 ± 0.11 mg GAE/g, 0.95 ± 0.021 mg QE/g, and 17.24 ± 0.91 mg UAE/g, respectively. Therefore, BME contains substantial amounts of carbohydrates, proteins, and triterpenoids.

**Figure 1:**
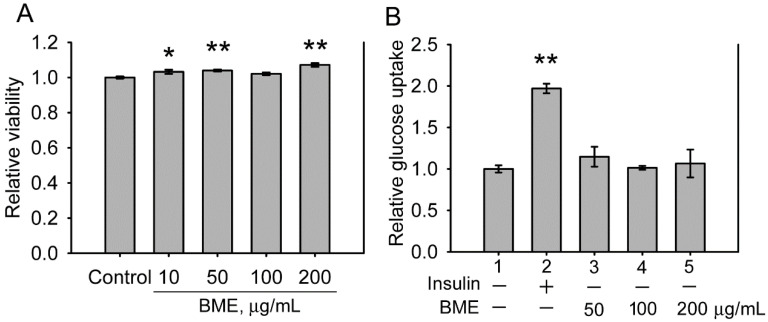
The effects of BME on normal IEC-18 cells. A, cytotoxicity assays. IEC-18 cells were treated with the solvent (control), 10, 50, 100 or 200 μg/mL BME for 6 h and subjected to a cell proliferation assay. B, glucose uptake assays in IEC-18 cells treated for 5 h with 100 nM insulin, 50, 100, or 200 μg/mL EPSs as indicated underneath the histogram. Relative glucose uptake versus Group 1 was determined. Both experiments were performed twice independently, each in triplicate. The data are the mean ± SE (N = 6). *P < 0.05 and **P < 0.005 versus the control (A) or Group 1 (B).

The cytotoxicity of BME to IEC-18 cells, an insulin-sensitive rat enterocyte cell line 19, was examined. Figure 1A displays that BME at 10-200 μg/mL is not toxic to IEC-18 cells. Therefore, the effect of BME on the glucose consumption of IEC-18 cells was characterized. Figure 1B reveals that insulin increased the glucose utilization of IEC-18 cells as reported previously 19. BME at 50-200 μg/mL did not show an obvious effect on promoting the glucose uptake of the cells, suggesting that BME does not possess an insulin-like effect to stimulate glucose intake by the enterocytes. Hence, BME does not affect the glucose consumption of normal enterocytes.

**Figure 2:**
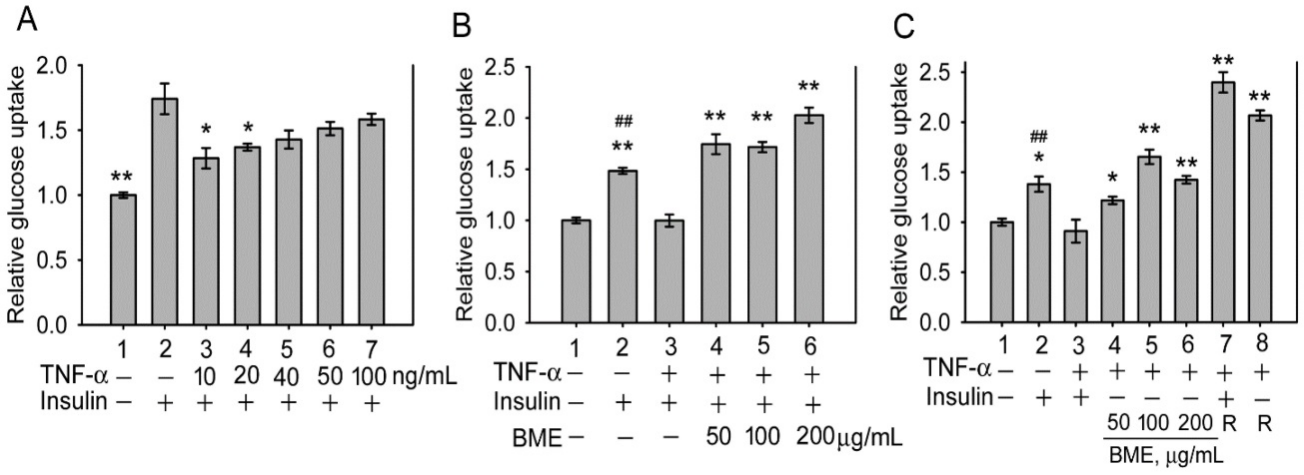
Glucose uptake assays in insulin-resistant IEC-18 cells. A**,** cells were pretreated with 10, 20, 40, 50 or 100 ng/mL TNF-α, followed by stimulation with 100 nM insulin. The experiment was performed in triplicate. B and C, cells were pretreated with 20 ng/mL TNF-α, followed by stimulation with 50, 100 or 200 μg/mL BME in the presence (B) or absence (C) of 100 nM insulin. In C, 50 μM rosiglitazone (R) was also tested as a positive control with (Group 7) or without (Group 8) insulin. Experiments were performed twice independently, each in triplicate. Relative glucose uptake versus Group 1 is shown. The data are the mean ± SE (N = 3 in A; N = 6 in B and C). *P < 0.05 and **P < 0.005 versus Group 2 (A) or Group 3 (B and C); ^ ##^*P* < 0.005 versus Group 1 (B and C).

**Figure 3:**
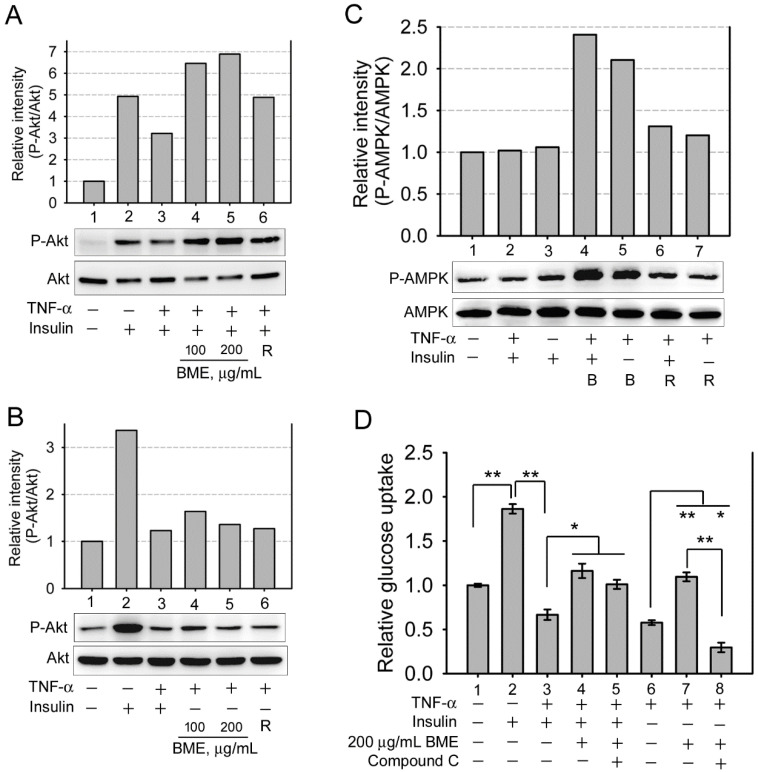
The effects of BME on Akt and AMPK. A, B, and C, western blot analysis of the levels of phosphorylated Akt, total Akt (A, B), and phosphorylated AMPK, total AMPK (C). IEC-18 cells were pretreated with 20 ng/mL TNF-α, followed by stimulation with 100 or 200 μg/mL BME, or 50 μM rosiglitazone (R) in the presence or absence of 100 nM insulin, as indicated underneath the blots. In C, the concentration of BME (indicated as B) was 200 μg/mL. Experiments were performed three times independently. The result from one of the experiments is shown. D, glucose uptake assays in IEC-18 cells. Cells were pretreated with 20 ng/mL TNF-α, followed by the treatment as indicated underneath the histogram. Experiments were performed in triplicate. The data are the mean ± SE (N = 3). *P < 0.05 and **P < 0.005 between the indicated groups.

Subsequently, the effect of BME on insulin-resistant cells was analyzed. Inflammatory cytokines such as TNF-α are critical factors causing insulin resistance of tissues 33-35. Figure 2A shows that pretreatment by TNF-α at 10 and 20 ng/mL effectively decreased insulin-promoted glucose uptake by IEC-18 cells (Groups 3 and 4 versus Group 2), suggesting the occurrence of insulin resistance in these cells, whereas higher concentrations of TNF-α did not show a higher efficacy (Groups 5-7). Thus, cells were pretreated with 20 ng/mL TNF-α, followed by insulin and BME co-stimulation. Figure 2B demonstrates that 50, 100, and 200 μg/mL BME all effectively enhanced the glucose uptake of TNF-α-pretreated cells (Groups 4-6) compared to the insulin-resistant control (Group 3), indicating that BME can promote the glucose consumption of insulin-resistant enterocytes. Subsequently, TNF-α-pretreated cells were stimulated by BME alone, without insulin. As shown in Figure 2C, BME at 50, 100, and 200 μg/mL (Groups 4, 5, 6) still significantly elevated the glucose uptake of the cells compared to the control (Group 3). Meanwhile, as a positive control, rosiglitazone, a thiazolidinedione-type medicine for treating diabetes, also promoted the glucose consumption of the cells in the presence or absence of insulin (Groups 7 and 8). Figure 2C reveals that BME alone, without insulin, can still enhance the glucose uptake of insulin-resistant cells. We define this function as insulin substitute herein. Therefore, the increased glucose consumption by the co-treatment of BME and insulin in Figure 2B might be irrelevant to the presence of insulin. Put differently, BME might not work as an insulin sensitizer. To clarify this issue, the effect of BME on Akt, an effector in the insulin-signaling pathway, was assayed. Figures 3A and 3B show that TNF-α decreased insulin-induced Akt activation (Lane 3 versus Lane 2), whereas the addition of 100 and 200 μg/mL BME recovered the elevation of Akt phosphorylation in the presence of insulin (Figure 3A, Lanes 4, 5), similar to the effect of rosiglitazone (Figure 3A, Lane 6). However, this effect of BME or rosiglitazone on Akt activation was not obvious in the absence of insulin (Figure 3B, Lanes 4, 5, 6). Therefore, BME alone could not activate Akt, yet it recovered insulin-promoted Akt activation in TNF-α-treated cells, indicating a function of insulin sensitizer. Together, Figures 2, 3A, and 3B suggest that BME was bi-functional, acting as an insulin sensitizer and an insulin substitute. Hence, the increased glucose uptake observed in insulin-resistant cells co-treated by BME and insulin (Figure 2B Groups 4-6) could be a result of the combined effects of insulin sensitizing and insulin substitution functions of BME. Meanwhile, data in Figure 3B also reveal that the insulin-substitute function of BME was not mediated by the activation of Akt, suggesting that it depends on other mechanisms to confer the insulin-substitute function.

Several natural compounds isolated from bitter melon were demonstrated to activate AMP-activated protein kinase (AMPK) 4, 8, which can increase glucose consumption of cells without insulin and without Akt activation. Therefore, whether BME activated AMPK in the enterocyte cell line was analyzed. Figure 3C manifests that 200 μg/mL BME obviously activated AMPK in the presence or absence of insulin in TNF-α-treated IEC-18 cells (Lanes 4 and 5), and 50 μM rosiglitazone showed a weaker effect (Lanes 6 and 7). Meanwhile, insulin did not obviously activate AMPK in this cell line (Lane 3), meaning that AMPK is probably not involved in insulin-mediated hypoglycemic function in this cell line. Moreover, Figure 3D shows that compound C, an AMPK inhibitor, did not significantly inhibit BME-promoted glucose uptake of insulin-resistant cells when insulin was present (Group 5 versus Group 4). This further supports that BME has a function of insulin sensitizer, which recovers the function of insulin, yet the function of insulin does not depend on AMPK in this cell line. Therefore, AMPK inhibitor did not obviously interfere with the glucose uptake of this group. On the contrary, compound C effectively suppressed BME-promoted glucose uptake of TNF-α-pretreated cells when insulin was absent (Figure 3D, Group 8 versus Group 7), suggesting that AMPK mediates the insulin substitute function of BME in the absence of insulin.

### Bitter melon extract stimulating GLP-1 secretion from the enteroendocrine cells

**Figure 4:**
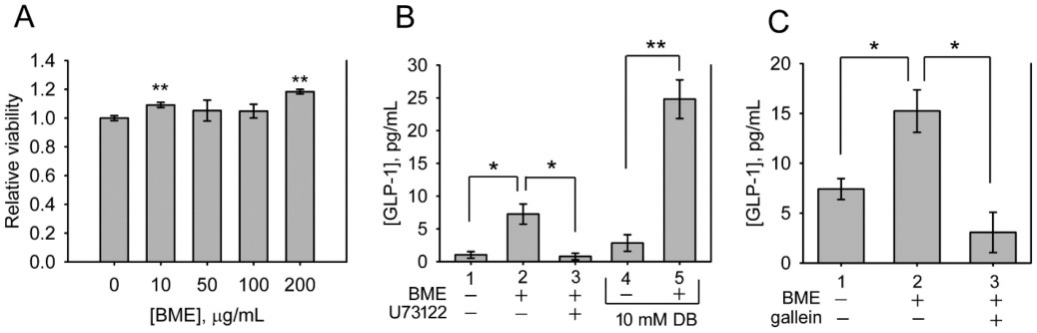
BME promoted the secretion of GLP-1 from NCI-H716 cells. A, cytotoxicity assays. NCI-H716 cells were treated with the solvent (0; control), 10, 50, 100 or 200 μg/mL BME for 6 h and subjected to a cell proliferation assay. Experiments were performed twice independently, each in triplicate. The data are the mean ± SE (N = 6). B and C, GLP-1 secretion assays. NCI-H716 cells were treated for 1 h with 200 μg/mL BME, 10 μM U73122, 10 μM gallein, 10 mM denatonium benzoate (DB), or the respective solvent, as indicated underneath the histogram. The culture medium was subjected to GLP-1 concentration assay by using an ELISA kit. Experiments were performed in quadruplicate (B) or in triplicate (C). The data are the mean ± SE. *P < 0.05 and **P < 0.005 versus the control (A) or between the indicated groups (B and C).

**Figure 5:**
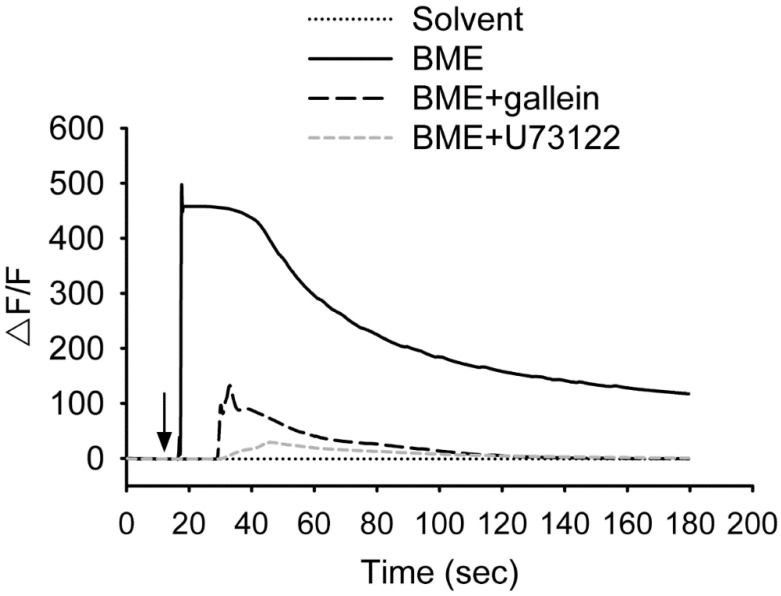
BME induced calcium responses in NCI-H716 cells. Cells were treated with 200 μg/mL BME or the solvent, or pretreated with 10 μM gallein or 10 μM U73122 for 30 min before the addition of 200 μg/mL BME, and subjected to calcium fluorescent imaging assays. The arrow indicates when BME or solvent was added to the medium. Experiments were performed three times independently. The result from one of the experiments is shown.

The bitterness of bitter melon indicates that the constituents of bitter melon activate TAS2Rs. Therefore, whether BME promotes the secretion of GLP-1 from enteroendocrine cells was analyzed. NCI-H716 cells, a human enteroendocrine cell line, were treated with 10-200 μg/mL BME to test cytotoxicity. Figure 4A shows that BME is not toxic to NCI-H716 cells. Subsequently, the secretion of GLP-1 from NCI-H716 was found to be enhanced while being treated with BME, as revealed in Figures 4B and 4C (Group 2 versus Group 1). Denatonium benzoate, a bitter compound demonstrated to elevate GLP-1 secretion, also increased GLP-1 secretion from the cells (Figure 4B, Group 5 versus Group 4). To evaluate whether the activation of TAS2R contributes to BME-induced GLP-1 secretion, the inhibitor of PLCβ_2_ (U73122) or Gβγ complex (gallein) was added. Consequently, U73122 (Figure 4B, Group 3) and gallein (Figure 4C, Group 3) both effectively suppressed BME-induced elevation of GLP-1 secretion. Furthermore, Figure 5 demonstrates that BME induced an obvious increase in intracellular [Ca^2+^], a biomarker of the activation of the TAS2R-signaling pathway. When cells were pre-treated with gallein or U73122, the BME-induced calcium response was apparently suppressed. Together, these data suggest that BME can effectively enhance GLP-1 secretion from enteroendocrine cells, which is likely mediated by the activation of the TAS2R-signaling pathway.

## Discussion

In this study, the extract of bitter melon exhibited three functions upon intestinal cells that are likely associated with its hypoglycemic effect. BME works as an insulin sensitizer and an insulin substitute against insulin-resistant enterocytes, and as a GLP-1 secretagogue on enteroendocrine cells. The insulin sensitizing function recovers insulin-induced activation of Akt. The insulin substitution function is likely due to the activation of AMPK, which mediates an insulin-independent increase of glucose uptake by the cells. The insulin sensitizing and the insulin substitution functions together re-boost glucose utilization by insulin-resistant enterocytes. The GLP-1 secretagogue function of BME may be rendered by the activation of TAS2Rs. These data support that BME has a hypoglycemic function on insulin-resistant or type 2 diabetic cases. Indeed, animal tests and at least some clinical trials have shown anti-diabetic effects of bitter melon on type 2 diabetic models or individuals 12, 36, 37, while the effect of bitter melon on the intestines may be one of the critical mechanisms underlying the anti-diabetic functions of bitter melon *in vivo*, but was not revealed, nor explored previously. On the other hand, BME did not show an insulin-like activity to increase the glucose consumption of normal enterocytes (Figure 1B). Thus, BME probably does not promote glucose utilization by normal tissues and may not be helpful in type 1 diabetic cases.

Bitter melon was reported to ameliorate insulin resistance 4, 11. Our data are consistent with this previous finding, owing to that BME exhibited an insulin-sensitizing function on insulin-resistant IEC-18 cells. This activity was revealed by that BME recovered Akt activation only in the presence of insulin in TNF-α-treated cells (Figure 3A and 3B). Moreover, in TNF-α-treated cells, the inhibition of AMPK suppressed the insulin substitute function (Figure 3D, Group 8), but did not inhibit the increased glucose uptake caused by the co-stimulation of BME and insulin (Figure 3D, Group 5). The latter further supports the existence of an insulin sensitizing activity of BME.

The findings that BME functions as an insulin sensitizer and an insulin substitute in insulin-resistant cells are consistent with the results of earlier studies using different cell types or tissues. Previously, bitter melon extract was shown to promote the glucose consumption of an insulin-resistant liver cell line 3, 4. Natural compounds isolated from the fruit and the stalk of* M. charantia* L. were reported to work as insulin sensitizers and insulin substitutes in insulin-resistant liver cell line 8. Bitter melon extract was also reported to improve the insulin sensitivity of skeletal muscle in high-fat-fed rats with signs of insulin resistance 38. Therefore, there is consistency between results obtained from cells of different tissues. In addition, one study showed that bitter melon natural compounds were transported across an intestinal epithelial cell layer, and some of them were absorbed inside the epithelial cells 10, stating that bitter melon compounds can interact with the intestines *in situ*, as well as with other tissues through circulation. Overall, our findings and others suggest that apart from being absorbed by the intestines and circulated to other tissues, bitter melon ingredients also interact with intestinal cells *in situ*, and the interactions are likely part of the mechanisms underlying the hypoglycemic function of bitter melon. The responses of the intestines to bitter melon likely work in concert with those of other tissues to result in the anti-diabetic effect of bitter melon.

In agreement with our findings that BME acts as GLP-1 secretagogue on enteroendocrine cells, bitter melon extract was shown recently to increase serum GLP-1 level in normal and diabetic rats 39. Several of the reported beneficial effects of bitter melon on glycemic control coincide with the functions of GLP-1. Bitter melon were shown to improve insulin resistance, increase insulin secretion in type 2 diabetic patients, as well as repair damaged β-cells 4, 11, 12, 36. These functions are consistent with several functions of GLP-1, which promotes glucose-dependent insulin secretion, increases insulin sensitivity of peripheral tissues, enhances insulin synthesis in β-cell, and promotes β-cell survival 40. Therefore, it is likely that some of the reported functional effects of bitter melon on glycemic control are mediated by GLP-1.

The mechanism underlying the insulin sensitizing effect of BME on insulin-resistant enterocytes was not elucidated in this study. However, insulin resistance is closely associated with inflammation, and type 2 diabetes is regarded as a low-grade, chronic inflammation-related disorder 34. Thus, the pro-inflammatory factor TNF-α obviously induced insulin resistance in IEC-18 cells. Earlier studies have demonstrated that bitter melon contain ingredients possessing anti-inflammatory activities 32, 41. Specifically, bitter melon' natural compounds were shown to suppress TNF-α-induced inflammation in a hepatic cell line 32, 42. Thus, we propose that the insulin sensitizing effect of BME on the TNF-α-treated enterocytes may be a result of its anti-inflammatory function, ameliorating inflammation-induced insulin resistance.

Our data suggest that BME activates the TAS2R-signaling pathway in enteroendocrine cells and leads to GLP-1 secretion. However, which isoforms of TAS2Rs are activated by BME is not clarified. There are 25 members in the human TAS2R family, and one molecule may activate more than one TAS2R isoform 26, 43. Considering the diversities of molecules contained in BME, it is expected that multiple isoforms of TAS2Rs are activated by BME. Using natural compounds isolated from bitter melon to identify the TAS2R isoforms being activated will provide more insightful information.

## Conclusion

In summary, BME contains components with insulin sensitizing, insulin substitution, and GLP-1 secretagogue functions upon intestinal cells. The exact principles of bitter melon possessing these functions deserve to be identified. Meanwhile, these findings suggest that apart from absorbing bitter melon ingredients from the lumen for transporting to other organs through circulation, the intestines may actively participate in the hypoglycemic mechanisms of bitter melon. The interactions of bitter melon compounds with the intestinal epithelial cells *in situ* are likely a significant part in the hypoglycemic action of bitter melon.

## Abbreviations

AMPK: AMP-activated protein kinase
BME: bitter melon extract
DB: denatonium benzoate
DPBS: Dulbecco's phosphate-buffered saline containing 2 mM calcium
GAE: gallic acid equivalents
GLP-1: glucagon-like peptide 1
IP3: inositol-1,4,5-triphosphate
PLCβ_2_: phospholipase C β_2_
QE: quercetin equivalents
TAS2R: bitter-taste receptor
UAE: ursolic acid equivalents
